# In Silico Screening and In Vitro Assessment of Natural Products with Anti-Virulence Activity against *Helicobacter pylori*

**DOI:** 10.3390/molecules27010020

**Published:** 2021-12-21

**Authors:** Maciej Spiegel, Paweł Krzyżek, Ewa Dworniczek, Ryszard Adamski, Zbigniew Sroka

**Affiliations:** 1 Department of Pharmacognosy and Herbal Medicines, Wroclaw Medical University, Borowska 211A, 50-556 Wroclaw, Poland; zbigniew.sroka@umw.edu.pl; 2 Department of Microbiology, Faculty of Medicine, Wroclaw Medical University, Chalubinskiego 4, 50-368 Wroclaw, Poland; ewa.dworniczek@umw.edu.pl; 3 Laboratory of Microscopic Techniques, Institute of Genetics and Microbiology, University of Wroclaw, Przybyszewskiego 63, 50-001 Wroclaw, Poland; ryszard.adamski@microb.uni.wroc.pl

**Keywords:** *Helicobacter pylori*, alternative therapy, phytotherapy, virulence, biofilm, coccoid forms, dioscin, molecular docking, Bioflux

## Abstract

*Helicobacter pylori* is one of the most frequent human pathogens and a leading etiological agent of various gastric diseases. As stringent response, coordinated by a SpoT protein, seems to be crucial for the survivability of *H. pylori*, the main goal of this article was to use in silico computational studies to find phytochemical compounds capable of binding to the active site of SpoT from *H. pylori* and confirm the ability of the most active candidates to interfere with the virulence of this bacterium through in vitro experiments. From 791 natural substances submitted for the virtual screening procedure, 10 were chosen and followed for further in vitro examinations. Among these, dioscin showed the most interesting parameters (the lowest MIC, the highest anti-biofilm activity in static conditions, and a relatively low stimulation of morphological transition into coccoids). Therefore, in the last part, we extended the research with a number of further experiments and observed the ability of dioscin to significantly reduce the formation of *H. pylori* biofilm under Bioflux-generated flow conditions and its capacity for additive enhancement of the antibacterial activity of all three commonly used antibiotics (clarithromycin, metronidazole, and levofloxacin). Based on these results, we suggest that dioscin may be an interesting candidate for new therapies targeting *H. pylori* survivability and virulence.

## 1. Introduction

The rapid spread of drug-resistant bacteria is now occurring all over the world, posing a threat to the forms and availability of currently known treatments [1,2,3]. This situation has worsened over time and now, according to many scientists and clinicians, medicine is gradually entering the post-antibiotic era [4,5]. The universality of antibiotic usage in various sectors of human life generates a selective pressure on human microflora and pathogens colonizing the human body [6]. Keeping in mind that the digestive system is the largest human reservoir of bacteria and is constantly subjected to the selection pressure to diverse antimicrobials, monitoring resistance and improving methods aimed at combating pathogens persistently colonizing this system is of paramount importance [7,8]. In this context, *Helicobacter pylori* seems to be one of the most important threats [9,10].

*H. pylori* is one of the most frequent human pathogens and a leading etiological agent of various gastric diseases, including gastritis, peptic ulcers, and gastric cancers (adenocarcinomas and mucosa-associated lymphoid tissue lymphomas) [11]. The success of *H. pylori* as a gastric pathogen is associated with its ability to survive in harsh conditions generated by the stomach [12]. Complex physiological processes related to virulence, such as biofilm formation [13,14,15] and morphological transformation [16,17,18], although described for first time for *H. pylori* a long time ago, just recently began to be shown as crucial to the survival of this microorganism and the relationship to therapeutic failures. This is reflected not only in the increasing amount of research conducted on both biofilms and coccoid forms of *H. pylori*, but also in recommendations encouraging efforts to intensify the search for methods effective in interfering with these mechanisms [13,16,19,20].

In the past few years, the relevance of computational chemistry tools as partners to experimental investigations has been emphasized. Among several application fields, computer aided drug design is the most promising and intensively utilized. These in silico studies focus on searching for possible drug candidates and have become a strategy of choice in examining and developing novel compounds with the desired activity due to their satisfying results [21]. Not only does this approach significantly reduce the time required to conduct studies by screening the entire library in a short period of time and thus discarding inactive compounds at the outset, but it also provides opportunities to discover novel pharmaceuticals that may be used in the treatment of certain diseases such as cancers [22] or neurodegenerative disorders [23]. This is especially true nowadays, as the methodology is being coupled with artificial intelligence, which improves its accuracy and, therefore, leads to new discoveries [24].

Plant substances are well known for their beneficial, health-promoting properties [25]. Studies show that phytochemicals have a multidirectional mechanism of action in their antibacterial activity: disruption of cytoplasmic membrane function and structure (including the efflux system), interaction with the membrane proteins (ATPases and others), interruption of DNA/RNA synthesis and function, destabilization of the proton motive force and induction of ion leakage, prevention of enzyme synthesis, coagulation of cytoplasmic constituents, and interruption of microbial communication [26]. Some publications highlight the potential of natural products to influence the survivability of *H. pylori*, either by reducing resistance to specific antibiotics [27] or by postponing biofilm development [28].

It seems that stringent response may be an excellent target in novel therapies fighting pathogenic microorganisms [29]. The stringent response is a phenomenon related to microbial reprogramming during which ribosomal synthesis is downregulated, while stress-associated genes are upregulated. This process is controlled by the intracellular concentrations of alarmon (guanosine tetra- and pentaphosphate [(p)ppGpp]), a molecule existing in most bacteria that is synthesized and degraded by RelA and SpoT, respectively. In the case of *H. pylori*, however, the stringent response is coordinated only by a one bifunctional SpoT protein regulating the level of this messenger substance [30,31]. Although the amount of data on SpoT of *H. pylori* is still very limited, studies by some research teams have observed that activity of SpoT is associated with stress and defense responses in *H. pylori*, and thus, interfering with its protective activity might be a promising way of fighting this pathogen [32,33,34].

The main goal of our research was to use in silico computational studies to find phytochemical compounds capable of binding to the active site of SpoT from *H. pylori* and to confirm the ability of the most active candidates to interfere with the virulence of this bacterium through in vitro experiments.

## 2. Results and Discussion

### 2.1. Virtual Screening

**Figure 1:**
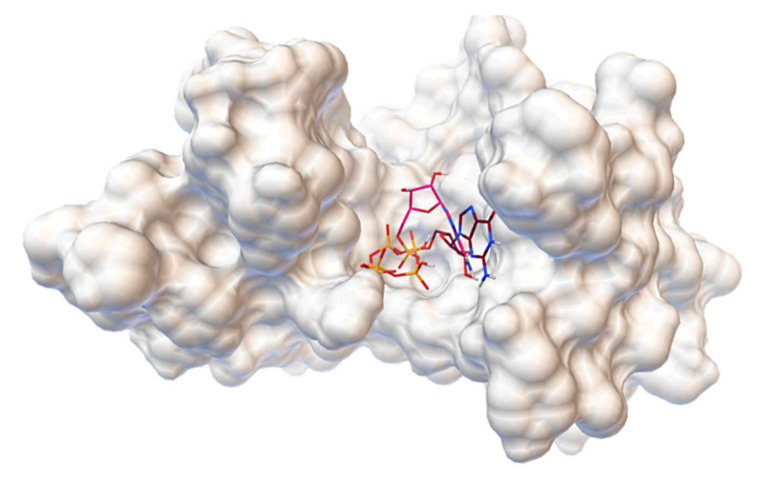
The 3D structure of SpoT domain exhibiting kinase activity with ATP and GTP bound to the common cavity.

We were able to evaluate binding sites and discover interactions that are likely to be relevant for possible competitive inhibitors by docking natural ligands, ATP and GTP, to the constructed structure of SpoT protein third domain. The ligands are located in the common cavity in the 3D visualization of the aforementioned process, which is shown in Figure 1. It should be noted that, despite the fact that the docking box was defined for the whole domain, the ligands appear only in the presented region, although in various conformations. Furthermore, with the exception of ATP’s ribose, the structures are in close proximity to one another, suggesting the potential for Pi transfer from ATP to GTP, which corresponds to diphosphokinase activity.

**Figure 2:**
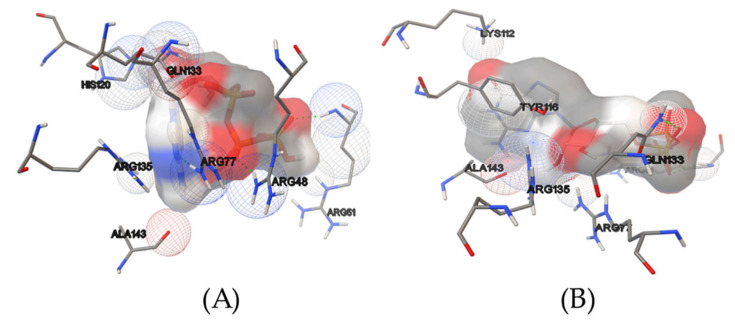
Amino acids involved in the binding of ATP (**A**) and GTP (**B**). Van der Waals interactions are depicted as wireframed spheres, with red representing oxygen, blue representing nitrogen, and gray representing carbon origin. The surfaces of the ligands are colored in the same way.

Figure 2 displays amino acids that interact with ligands and thereby contribute to their binding. Both natural ligands are surrounded by ARG77, ARG51, GLN133, ARG135, and ALA143 amino acids. Hydrogen bonds are formed with ATP via ARG51, ARG77, and ARG48, and with GTP via ARG51 and GLN133. Furthermore, a slew of van der Waals interactions can be seen, which are known to maintain ligands within the pocket.

To choose the compounds to be submitted for the experimental part of the research, the following approach was adopted. To assert the substance’s competitive nature, the preliminary screening results were filtered out of compounds having binding free energies of less than −7.6 kcal/mol, which corresponds to that of GTP (ATP has a binding free energy of −6.4 kcal/mol). They are listed in Table 1, and the whole list is available in Table S1. Furthermore, inhibition constants (*K*_i_) were calculated, as described in the Section 3. This preliminary screening yielded 105 of the most promising candidates, from which 10 hits were selected based on the following criteria: (1) comparably low binding free energies, (2) amino acid composition interacting similarly to those previously found for natural ligands, (3) visible structural differences, and (4) negligible toxicity to humans. A toxicity profile was established only for selected compounds with favorable parameters (e.g., comparably low binding free energies and/or the ability to interact with a high amount of amino acids in the target site of SpoT in a way similar to those found for natural ligands). The literature was searched for the toxicity data of these compounds (Table S2).

### 2.2. MICs

In the first step, we decided to determine the antimicrobial activity of 10 previously selected compounds by determining their minimal inhibitory concentration (MIC) values (Table 2). We noticed that dioscin (MIC = 64 µg/mL) was the most active one, followed by amentoflavone and hypericin (both with MIC = 128 µg/mL), and aescin (MIC = 256 µg/mL). The remaining six compounds showed no antibacterial activity against *H. pylori* J99 in the concentration range used in this study (MIC > 256 µg/mL). Despite numerous studies on the anti-inflammatory [35,36,37] and antitumor [38,39,40,41] activity of dioscin or diosgenin (an aglycone form of dioscin), the data on their antimicrobial action are very scarce. Among the few articles available, three concerning fungi [42,43,44] and one indicating activity against anaerobic bacteria [45] could be found. For this reason, we believe that the results obtained by our team bring the aspect of novelty in the context of using dioscin in anti-bacterial therapy.

### 2.3. Anti-Biofilm Activity

To obtain a more complete picture of the activity of the tested compounds against *H. pylori*, we extended our screening to include the determination of anti-biofilm activity and the effect on bacterial morphology. Both biofilm formation [13,14,15] and transformation into coccoid morphotype [16,17,18] are phenomena related to the tolerance of *H. pylori* to antimicrobial substances. Therefore, we assumed that ideally the tested compounds should be able to reduce the amount of biofilm without or with minimal stimulation of the spiral-to-coccoid transition (coccoid forms arise often intensively during exposure to antibiotics or environmental conditions unfavorable to bacterial physiology [16,17,18]).

**Figure 3:**
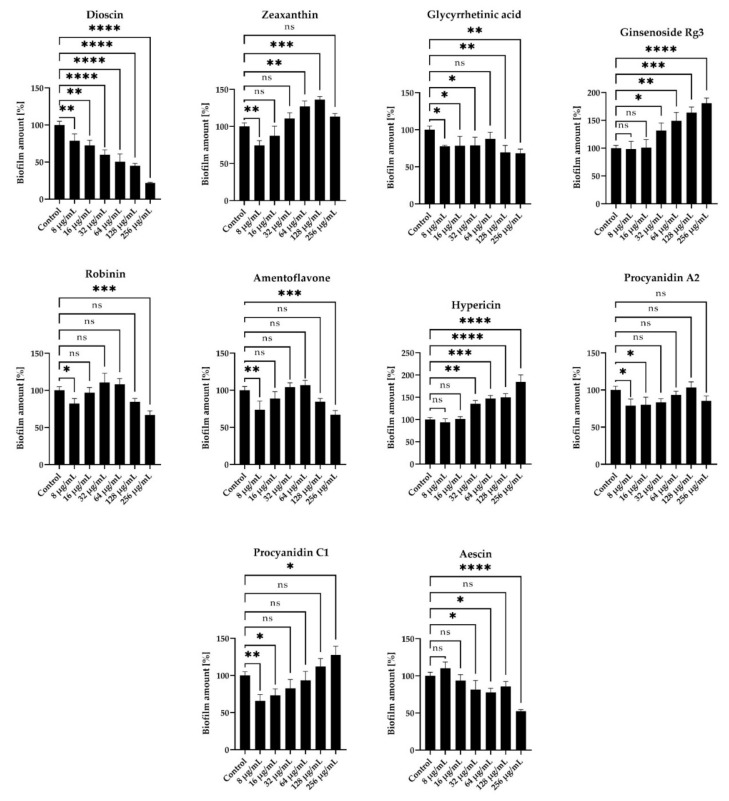
Anti-biofilm activity of 10 selected compounds of natural origin against *H. pylori* J99. The amount of biofilm under stationary conditions was determined using the crystal violet staining method. The study was performed with triplicate biological replications (*n* = 3). The *p*-value represented by ns, *, **, ***, and **** is equal to > 0.05, < 0.05, < 0.01, < 0.001, and < 0.0001, respectively.

We observed that dioscin was characterized by the most intense inhibitory activity against biofilm formation under static conditions, i.e., MICs reduced this process by about half (up to 50.2 ± 10.6%) (Figure 3). Importantly, at these concentrations, we detected a low level of coccoid *H. pylori* forms (approx. 20%) (Figure S1). Other compounds at MIC values for which we noticed an ability to decrease the amount of *H. pylori* biofilm include aescin (decrease to 52.3 ± 2.2%) or amentoflavone (decrease to 84.5 ± 4.4%) (Figure 3); however, for both of them, the number of coccoid forms was unsatisfactorily high (40–60%) (Figure S1).

In line with our earlier deduction, which indicated only a few publications describing the antimicrobial activity of dioscin, in this case also, it was difficult for us to refer to other data in the literature. Despite this, in the original article by Yang et al. (2018) [43], the ability of dioscin to interfere with a number of virulence factors of fungi from the *Candida* genus has been proved. This mechanism covered not only their biofilm formation, but also morphological transformation (transition to filamentous forms) and secretion of lytic exoenzymes. This seemed to confirm our primary assumptions about the possibility of using dioscin in anti-virulence therapy against *H. pylori*.

Surprisingly, for some substances—hypericin, ginsenoside Rg3, procyanidin C1, and zeaxanthin—we noticed a stimulating effect on the amount of *H. pylori* biofilm. For example, for hypericin at a concentration equal to 2 × MIC (256 µg/mL) the amount of biofilm increased to 184.4 ± 16% compared to the control (Figure 3). In our opinion, these observations carry great research and clinical implications. They indicate that the primary selection of antimicrobials based only on the MIC measurement may be insufficient or even confusing (hypericin, next to amentoflavone, as the second most active antimicrobial substance in this study) and emphasize the need to determine additional antimicrobial parameters [46]. Moreover, the above observations throw a shadow on the widespread hypothesis that microorganisms are unable to generate resistance or activate defense mechanisms against plant extracts or plant-derived compounds, in agreement with others [47,48,49]. On the other hand, it has been shown by several research groups that the activity of phytochemicals may be modulated by a concentration-dependent formation of dimers/trimers/polymers and aggregation-related changes in the antimicrobial action of these molecules [50,51,52].

**Figure 4:**
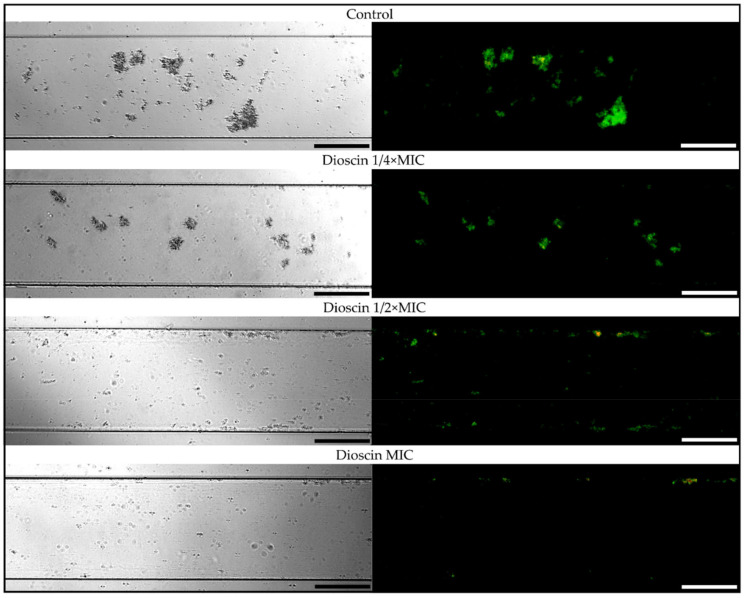
Representative photos obtained after 24 h of experiments determining anti-biofilm activity of dioscin under flow conditions against *H. pylori* J99. The flow conditions were generated using the Bioflux 1000 system. The MIC, ½ × MIC, and ¼ × MIC had values of 64 µg/mL, 32 µg/mL, and 16 µg/mL, respectively. Control samples were bacteria not exposed to any concentration of dioscin. The pictures on the right show cells stained with the LIVE/DEAD kit (green and red fluorescence showing live and dead cells, respectively) and viewed under a fluorescence microscope, while the pictures on the left show cells under a light microscope. Scale bars show 10 µm.

**Figure 5:**
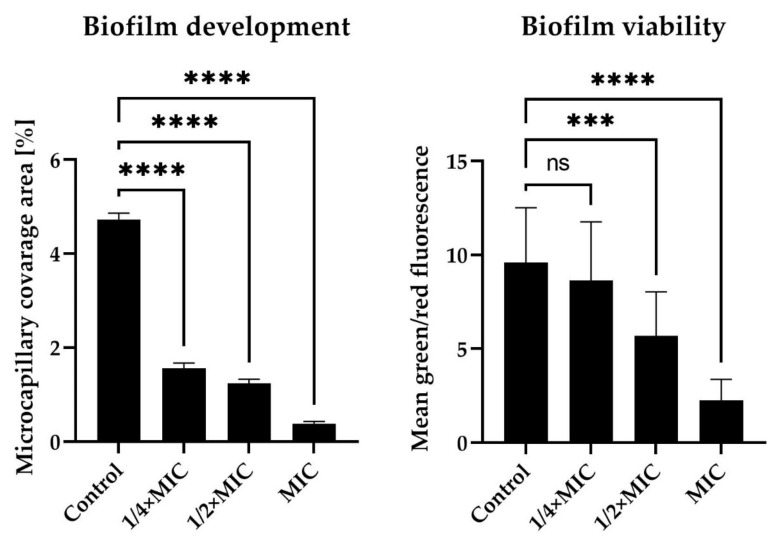
Data quantifying anti-biofilm activity of dioscin against *H. pylori* J99 after 24-h incubation under flow conditions. The flow conditions were generated using the Bioflux 1000 system. The MIC, ½ × MIC, and ¼ × MIC had values of 64 µg/mL, 32 µg/mL, and 16 µg/mL, respectively. Control samples were bacteria not exposed to any concentration of dioscin. Viability was defined as the ratio of green to red fluorescence of bacterial cells stained with the LIVE/DEAD kit, while the development of biofilm was verified by determining the degree of capillary coverage (both parameters were determined using the Bioflux Montage software). The study was performed with triplicate biological replications (*n* = 3). The *p*-value represented by ns, ***, and **** is equal to > 0.05, < 0.001, and < 0.0001, respectively.

Based on the most favorable parameters obtained for dioscin (the lowest MIC, the highest anti-biofilm activity in static conditions, and a relatively low stimulation of morphological transition into coccoids), we decided to extend the panel of studies on dioscin with the verification of its anti-biofilm activity against *H. pylori* J99 under flow conditions. For this purpose, we used an automatic system, namely Bioflux 1000, generating the flow of culture medium and enabling observation of microbes in real time [53]. Although this equipment has previously been used to determine the antibacterial and/or anti-biofilm activity of antibiotics and newly tested compounds against different bacterial genera [54,55,56], our team is the first to apply it to *H. pylori* (previously [57] and hereby). Analysis of time-lapse microscopy recordings of bacteria exposed to dioscin allowed us to confirm the concentration-dependent anti-biofilm effect of this compound (Figure 4, Figure 5 and Figure S2). After a 24-h incubation of *H. pylori* under the above flow conditions, we noticed a significant reduction in the amount of biofilm formed (4.72 ± 0.14% vs. 0.38 ± 0.05% of the capillary coverage in the control and MIC-exposed samples, respectively) (Figure 4 and Figure 5). Moreover, we observed a decrease in the green/red fluorescence ratio of dioscin-treated bacterial cells (9.59 ± 1.17 vs. 2.26 ± 0.11 for the control and MIC-treated samples, respectively) (Figure 4 and Figure 5).

### 2.4. Morphostructural Analysis of Bacterial Cells

**Figure 6:**
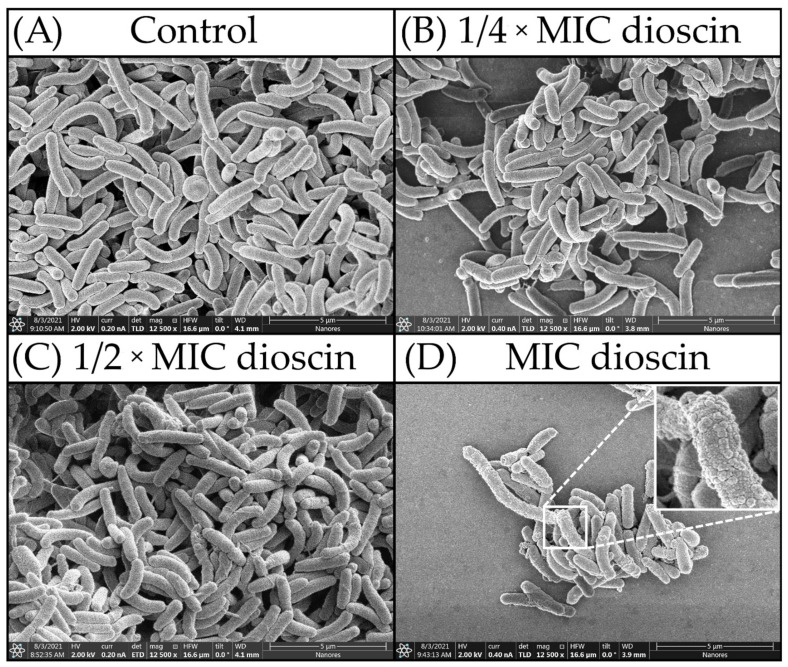
SEM micrographs of *H. pylori* cells: (**A**) untreated cells (control); (**B**) cells treated with ¼ × MIC of dioscin; (**C**) cells treated with a ½ × MIC of dioscin; (**D**) cells treated with a MIC of dioscin.

To confirm our assumptions about the lack of degradation of *H. pylori* cells exposed to concentrations of ¼ × MIC—MIC of dioscin, we performed morphostructural analysis of bacterial cells using scanning electron microscopy (SEM). The results obtained from the micrographs were unequivocal (Figure 6), and in all cases the bacterial cells were unchanged in size. *H. pylori* cells treated with dioscin at ¼ × MIC, ½ × MIC, and MIC concentrations showed normal morphology (Figure 6B–D), where they presented typically spiral/rod forms and non-numerous coccoids (both morphological forms were with intact cell surface). Similarly, images of the untreated control cells showed undeformed structure of their cell membrane (Figure 6A). It is noteworthy that the cells treated with a MIC value of dioscin displayed several apparent, distinguished signs showcasing a specific defense mechanism of *H. pylori* expressed in secretion of membrane vesicles (Figure 6D).

Membrane vesicles are nanometric structures that are secreted by both Gram-positive and Gram-negative bacteria [58,59,60]. Historically, they have been considered as evidence of microbial cell membrane instability and lysis. Nowadays, however, it has been proved that the increase in secretion of these structures is associated with adaptation to unfavorable environmental conditions, as they may promote cell–cell interactions and biofilm development [61]. For diverse groups of bacteria, an intensification in the production of membrane vesicles has been observed in response to exposure of bacteria to different classes of antibiotics and other antimicrobial compounds [62]. For *H. pylori*, also, it has been shown that treatment with antibiotics (including clarithromycin and levofloxacin) increases the amount of membrane vesicles with a positive effect on their survival in these conditions [63]. On this basis, we suspect that at MIC levels, dioscin may also induce a similar mechanism in *H. pylori* cells. However, this type of research is beyond the main scope of this research article, and the hypothesis we presented will require verification in the future.

Summarizing this part of the analysis, we did not observe any significant structural changes in cells treated with dioscin regardless of the applied concentrations. This led us to believe that the observed anti-biofilm activity of dioscin did not depend directly on the destruction of *H. pylori* cells and that a different mechanism was responsible for the death of the bacteria.

### 2.5. Checkerboard Assay

**Figure 7:**
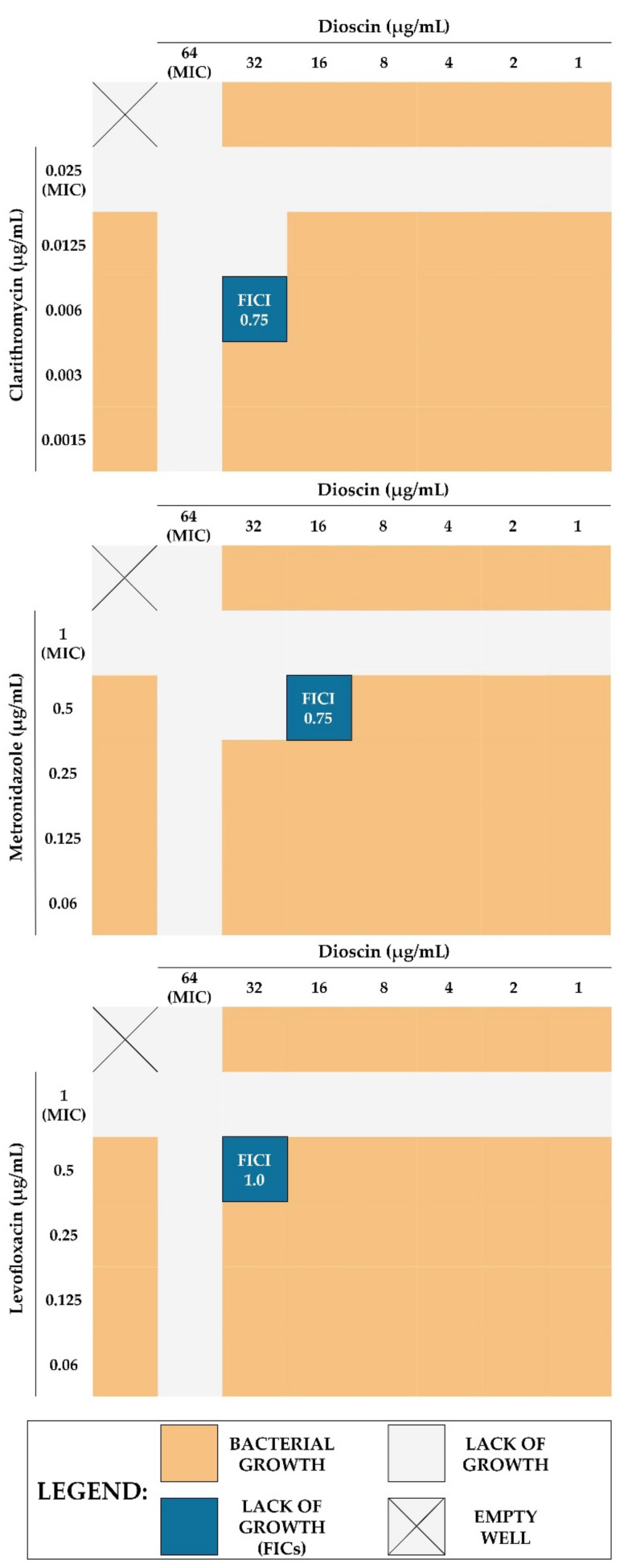
Graphics showing the interactions between dioscin and the three classically used antibiotics (clarithromycin [CLR], metronidazole [MTZ], and levofloxacin [LEV]) in antibacterial activity against *H. pylori* J99. Interactions were determined using the checkerboard assay. FICI values of ≤ 0.5, > 0.5 to ≤ 1, and > 1 to ≤ 4 were expressed as synergistic, additive, and neutral, respectively.

Next, we decided to confirm our proof-of-concept for the usefulness of dioscin in *H. pylori* therapy. In this context, using the checkerboard method, we determined the types of interactions between dioscin and three antibiotics classically used in *H. pylori* treatment (clarithromycin [CLR], metronidazole [MTZ], and levofloxacin [LEV] [64]). For all of them, the existence of additive interactions was demonstrated (FICI = 0.75 for both CLR and MTZ, and FICI = 1.0 for LEV), allowing us to reduce the concentrations of the tested components by 2–4 times (Table 3 and Figure 7). At this point, it is worth mentioning that this effect could be potentially stronger in relation to antibiotic-resistant strains of *H. pylori*; however, the necessity to use a genomically characterized reference strain in these studies forced us to choose an antibiotic-sensitive strain. As we noticed positive interactions in anti-*H. pylori* activity for all tested antibiotics when combined with dioscin, we suspected that the mechanism of sensitizing bacterial cells to these antibiotics could be non-specific and related rather to the anti-virulent activity of dioscin, i.e., reduction of autoaggregation and biofilm formation or production of antibiotic-tolerant coccoid forms.

### 2.6. Concluding Computational Studies

Among the compounds tested, dioscin was shown to be a very promising candidate for reducing *H. pylori* biofilm formation and sensitizing to certain antibiotics. This substance was shown to be the most promising during molecular docking experiments since it had one of the lowest binding free energies of −9.8 kcal/mol. Only madecassoside was discovered to have a lower value (−10.6 kcal/mol) and shall be considered for further studies as a potent SpoT inhibitor.

**Figure 8:**
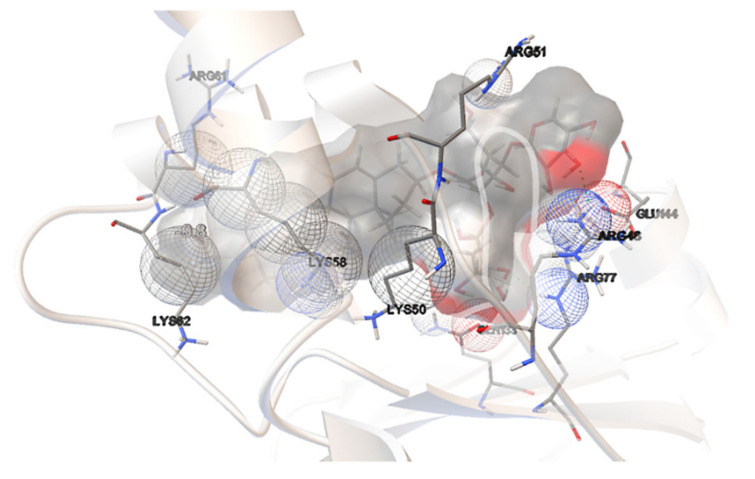
Dioscin interactions with the binding pocket of a SpoT protein of *H. pylori*.

Figure 8 illustrates our hit’s interactions with the binding pocket, using coloring schemes adequate to those discussed earlier. The hit and natural ligands shared four amino acids: ARG77, ARG61, GLN133, and ARG48. Although just two hydrogen bonds, interacting with dioscin saccharides, have been discovered in this binding mechanism, they arise from the amino groups of ARG48 and ARG77, just as they do for ATP and GTP, suggesting their relevance for the binding mode. Instead, we observed a number of van der Waals interactions, which underlined that the binding mechanism is strongly hydrophobic.

### 2.7. Limitations and Future Perspectives

In the last part of this article, we would like to highlight some limitations of our research. The results of molecular docking on the inhibition of SpoT from *H. pylori* by selected phytochemical compounds have been confirmed in experiments with indirect methods. The inclusion of these methods was related to the supervisory function of SpoT in the survivability of *H. pylori*, i.e., biofilm formation, morphological transformation, and antibiotic resistance. The capacity of dioscin to interfere with the above processes was demonstrated by reducing the amount of biofilm under stationary conditions (a crystal violet-based staining) and flow conditions (a microfluidic Bioflux system), low stimulation of coccoid formation (light and electron microscopy), and enhancement of the antibiotics’ activity (checkerboard assays). To directly confirm the ability of dioscin to inhibit SpoT of *H. pylori*, we should finally extend our research to the purified protein and isothermal titration calorimetry assays, which we intend to do in the future. In our opinion, this article may be an important voice in raising the scientific community’s awareness to the potential of SpoT in promoting *H. pylori* survival. The first reports on SpoT of *H. pylori* were published relatively long ago—2006 [30,31], but the continuation of research on this issue was averted for over a decade, and just in recent years has slowly reappeared [32,33,34]. We hope that the results presented in the current article, although preliminary, may help in the search for alternative therapies to combat the virulence of this pathogen.

## 3. Materials and Methods

### 3.1. Computational Analysis

#### 3.1.1. Bioinformatic Protocol

Due to the lack of the crystal structure of SpoT protein for any *H. pylori* strain at the time of writing this paper, a homology modeling pathway had to be undertaken. In the first step, an ATCC Genome Portal entry corresponding to the J99/ATCC 700824 strain [65] used within this study was thoroughly searched for the sequence encoding the SpoT protein. Since none of the identified product was classified as such, the UniProt database [66] was explored for the known sequence responsible for the aforementioned activity. The found entry (O25466), corresponding to the HP_0775 gene in *H. pylori* (strain ATCC 700392/26695) [67], was submitted for BLAST procedure [68], using BLOSUM62 matrix [69], against the genome of J99, yielding a sequence described by the satisfying results of 98% identity, 99% positivity, and 0% gaps.

It was trimmed out and used as an input for ThreaDomEx [70] to establish domains. With the cutoff of 0.56, five were identified, and each was submitted for I-TASSER [71,72,73,74], known for its continuous success in CASP experiments [75], to predict their structure and the activity resulting from it. Based on the anticipated GO:Scores, we decided to pick the first model of the third domain for further studies as the only one possessing a positive and satisfying C-Score (1.24), and the only one expressing kinase activity (GO:0016301).

**Figure 9:**
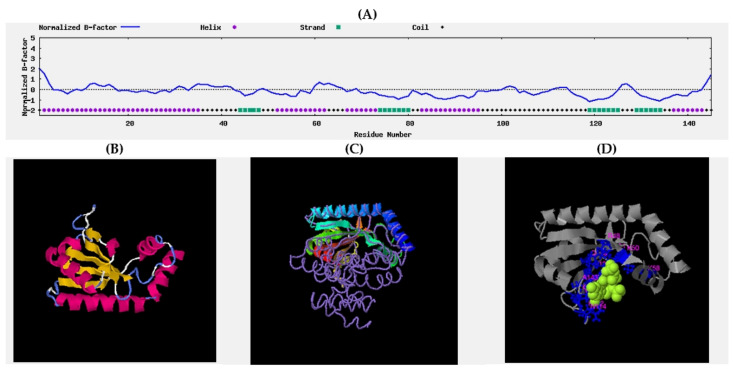
Supporting graphs: (**A**) B-factor plot; (**B**) homology model; (**C**) structure superposition of modeled protein (cartoon) and best hit—1vj7A (coil); (**D**) predicted ligand binding site.

The predicted secondary structure of the protein was satisfying, both with a confidence score average of 7.5 and a predicted normalized B-factor (Figure 9) [76,77] mostly below 0, which indicated that most of the residues are relatively stable in the structure. Three entries were found in the top 10 threading templates: 1vj7A (6 times), 5iqrA (2 times), and 5kpvA (2 times), and their corresponding Z-scores of 4.43, 4.09, and 3.32 indicated very good alignment between sequences. Importantly, 1vj7A represents a bifunctional catalytic fragment of RelSeq, the RelA/SpoT homolog from *Streptococcus equisimilis*. Four final tertiary structures were predicted, but only the first one represented a satisfying C-score parameter of 1.24, whereas the range may vary from −5 to 2, thus pointing to its satisfyingly correct global topology; corresponding to a TM-score equaling 0.88 ± 0.07 and a corresponding RMSD = 2.3 + 1.8 Å.

Apart from the structural features, function was also established. Although the highest C-score was only 0.40, the corresponding ligand was found to be guanosine 5′-(tetrahydrogen triphosphate) 3′-(trihydrogen diphosphate). This corresponds with the established EC numbers that represent activities of GTP diphosphokinase and guanosine-3′,5′-bis(diphosphate) 3′-diphosphatase and a GO:Score of 0016301 as one expressing kinase activity (C-score of all of these was 0.450).

#### 3.1.2. Molecular Dynamics

The obtained domain was submitted for MD simulation using Gromacs 2019.4. [78]. Its topology was prepared with CHARMM36m forcefield [79]. The domain was centered in a rectangular box, with 10.0 Å distance from the edge, and surrounded by TIP3 water molecules. Next, the system was neutralized with Na+ and Cl- ions placed randomly using the Monte Carlo algorithm [80]. The temperature of the system was set to 310.15 K (37 °C) to resemble that in the stomach.

Energy minimization was performed using the steepest descend algorithm. The minimized system was then submitted for NVT ensemble equilibration, which was carried out at 125 ps and 310.15 K temperature using a Nose-Hoover thermostat [81,82]. The production was performed in 1 ns of NPT ensemble, with the temperature controlled as previously, and pressure maintained at 1 bar with Parrinello-Rahman pressure coupling [83].

At each step, electrostatic interactions were subjected to the Particle Mesh Ewald method [84] with a cut-off value of 1.2 Å; the same cut-off value was set for Van der Waals interactions. The LINCS algorithm was used to convert bonds with hydrogen atoms to constraints [85].

#### 3.1.3. Virtual Screening

Due to the unknown active site, it was necessary to evaluate where the natural ligands (ATP and GTP) bind [86,87]. Firstly, a conformational search procedure was utilized in a molecular dynamic framework implemented in Gabedit [88], and three conformers of each of these molecules were obtained. These were submitted for optimization and frequency calculations in Gaussian quantum chemistry software package (revision D.01) [89], using density functional theory method B3LYP in 6-311++G(d,p) basis set, accompanied with GD3 dispersion correction [90] and SMD water solvent [91]. No imaginary frequency was present. The lowest one for each was chosen for the molecular docking procedure performed with Autodock Vina [92]. The water molecules were removed from the MD output and Gasteiger chargers added. Grid box size was set to cover the entire domain, and the docking procedure was started.

Having detected the eventual binding site, the grid box was moved and resized to cover it. Afterwards, an in-house library consisting of 791 natural substances belonging to the collection of phytochemicals from the Department of Pharmacognosy and Herbal Medicines, Wroclaw Medical University, Poland was submitted for a virtual screening procedure, generating a list of the most probable inhibitors.

The inhibition constant (*Ki*) was obtained from the binding free energy of the compound (ΔG) using the equation:

*Ki* = *exp*(Δ*G*/*RT*)

where, R is the gaseous constant (1.987 cal K^−1^ mol^−1^) and T is the temperature (310.15 K).

### 3.2. Laboratory Analysis

#### 3.2.1. Storage and Revival of Bacteria

The research was conducted using the reference *H. pylori* J99 (ATCC 700824) strain, obtained by courtesy of Dr. Anna Pawilk from Hirszfeld Institute of Immunology and Experimental Therapy, Polish Academy of Sciences. Bacteria were stored in Tryptic Soy Broth (TSB; Oxoid, Dardilly, France) supplemented with 15% glycerol at −70 °C [93,94]. After thawing, the tested bacterial strain was cultured on Columbia agar (Difco, Lublin, Poland) with 7% horse blood. The bacteria were grown for 3 to 5 days at 37 °C in a microaerophilic atmosphere (Genbox microaer kits, BioMerieux, Marcy-l’Étoile, France), and eventually sub-cultured again in the same conditions if needed.

#### 3.2.2. Antibacterial Activity Determination

The designation of antibacterial activity of 10 selected, plant-originated compounds (dioscin, aescin, glycyrrhetinic acid, ginsenoside Rg3, procyanidin A2 and C1, hypericin, robinin, amentoflavone, and zeaxanthin; all from Sigma-Aldrich, St. Louis, MO, USA) against *H. pylori* J99 was made by checking their minimum inhibitory concentrations (MICs) [93,94]. To obtain this, bacteria in a density of 10^7^ CFU/mL were cultured in 12-well titration plates (Bionovo, Legnica, Poland) filled with 1 mL of Brain Heart Infusion broth (BHI; Oxoid, Dardilly, France) with 5% fetal calf serum (FCS; Gibco, Paisley, Scotland, UK) and a concentration gradient of the tested compounds (2–256 µg/mL). The titration plates were then followed for 3 days of microaerophilic incubation at 37 °C and 100 rpm shaking (MaxQ 6000, ThermoFisher, Waltham, MA, USA). Then, bacterial turbidity in each well of the plates was determined visually, and the lowest concentration with the lack of growth was considered as the MIC.

#### 3.2.3. Impact on the Morphology and/or Morphostructure

The screening determination of the effect of high concentrations (64–256 µg/mL) of all 10 tested compounds on the *H. pylori* morphology was performed using light microscopy combined with a Gram-staining [95]. For each sample, bacterial suspension in a volume of 50 μL was dropped onto a coverslip and stained by Gram’s method. From each of the obtained preparations, 100 cells from three to five different view pools were counted and classified as spiral/rod-shaped or coccoid forms. Observations were made under the Olympus BX50 light microscope (Olympus Optical, Tokyo, Japan), using a 100× oil immersion objective with numerical aperture of 1.3.

In order to more accurately define the impact of dioscin on *H. pylori* J99 cells, bacteria were exposed to different concentrations of the compound and their morphostructure was visualized by scanning electron microscopy [95]. For this purpose, each well of the 12-well titration plates was filled with 1 mL BHI broth with 5% fetal calf serum containing 16 µg/mL, 32 µg/mL, and 64 µg/mL of dioscin (representing ¼ × MIC, ½ × MIC and MIC, respectively) and inoculated with *H. pylori* (10^7^ CFU/mL). Such plates were then followed for a 72-h incubation period at 37 °C under microaerophilic conditions. Untreated bacteria were a positive control. After incubation, each bacterial culture was centrifugated, and the pellets were fixed with 4% glutaraldehyde and post-fixed in 2% OsO4. Fixed samples were dehydrated in ethanol-acetone series and, air-dried, and a silver coating was sputtered on the bacteria surface, providing optimal conductivity. SEM microphotographs were obtained by a DualBeam SEM/Xe-PFIB FEI Helios G4 PFIB CXe microscope (Thermo Fisher Scientific, Eindhoven, Netherlands).

#### 3.2.4. Anti-Biofilm Activity Determination

##### Activity in Static Conditions

To assess the anti-biofilm activity of all 10 tested compounds against *H. pylori* J99, the crystal violet staining method was applied [96]. Bacteria in a final density of 10^7^ CFU/mL were suspended in 1 mL of BHI broth with 5% fetal calf serum and a gradient concentration (2–256 µg/mL) of the tested compounds, and were cultured for 3 days under microaerophilic atmosphere, 37 °C, and 50 rpm shaking. Then, the entire bacterial suspension was removed from each well, and the plates were dried and stained with a 0.1% crystal violet solution (Sigma-Aldrich, St. Louis, MO, USA) for 15–30 min. The dye was removed, and the wells were then rinsed twice with a phosphate buffer solution (PBS; Sigma-Aldrich, St. Louis, MO, USA) and flushed with 1 mL of 96% ethanol (Stanlab, Lublin, Poland). Two hundred microliters of crystal violet-stained alcohol was withdrawn from each well, transferred to a 96-well microtiter plate (Bionovo, Legnica, Poland), and measured for absorbance at OD_590_ with an Asys UVM 340 microplate reader (Biochrom Ltd., Cambridge, UK). The negative control consisted of wells without bacteria (the absorbance of these samples was used each time to subtract background from the tested samples).

##### Activity in the Flow Conditions

Confirmation of the anti-biofilm activity of dioscin against *H. pylori* J99 was performed under flow conditions created by the Bioflux 1000 system (Fluxion, San Francisco, CA, USA) [57]. To generate these conditions, 48-well low-shear plates composed of inlet and outlet wells connected by a capillary (Fluxion, San Francisco, CA, USA) were used. In the first stage, inlet wells were filled with 900 µL of BHI broth with 5% fetal calf serum and the desired concentration of dioscin (16 µg/mL, 32 µg/mL, and 64 µg/mL, representing ¼ × MIC, ½ × MIC, and MIC, respectively). Wells without dioscin served as a positive control. In the next stage, the above-mentioned medium was flowed through capillaries with a pressure of 10 dyne/cm^2^ for 10 sec, allowing components of the medium to cover the capillaries’ surfaces. Then, 100 µL of bacterial suspension (10^8^ CFU/mL) was placed into inlet wells to obtain the final bacterial density of 10^7^ CFU/mL in each well. A 1-day flow of the medium at 37 °C and microaerophilic atmosphere (Pecon Incubator XL S1, Carl Zeiss, Jena, Germany) was then directed from the input to the output wells with a pressure of 0.1 dyne/cm^2^. Following this, the flow of medium was interrupted, and the remaining medium from inlet wells was taken. The emptied inlet wells were filled with 100 µL of dye solution made from a LIVE/DEAD kit (Thermo Fisher, Waltham, MA, USA). The flow of 0.1 dyne/cm^2^ was turned on again to provide a 1-h staining of bacterial microcolonies and biofilms. After this, photos from capillaries were obtained with an inverted Axio Observer 7 microscope (GmbH, Carl Zeiss, Jena, Germany). Both the degree of capillary overgrowth and the ratio of green/red fluorescence (the ratio of live/dead bacteria) was determined using the Bioflux Montage software (Fluxion, San Francisco, CA, USA).

#### 3.2.5. Checkerboard Assays

The determination of interactions between dioscin and the most commonly used antibiotics (clarithromycin, metronidazole, and levofloxacin; all from Sigma-Aldrich, St. Louis, MO, USA) was achieved with checkerboard assays [96]. All experiments were carried out in four combined 12-well plates, creating a 48-well panel. Each well of these plates was loaded with 1 mL of BHI broth with 5% fetal calf serum, a bacterial suspension of 10^7^ CFU/mL, and a mixture of both tested substances (dioscin and one of the above-mentioned antibiotics). The concentration gradients of antibiotics (MIC to 1/16 × MIC) and dioscin (MIC to 1/64 × MIC) were generated in the vertical and horizontal direction, respectively. Plates with such content were followed for a 3-day period of microaerophilic culture at 37 °C with 100 rpm shaking. The interactions between dioscin and each of the antibiotics were checked by calculating a fractional inhibitory concentration index (FICI). The FICIs with values of ≤ 0.5, > 0.5 to ≤ 1, and > 1 to ≤ 4 were expressed as synergistic, additive, and neutral, respectively [27].

#### 3.2.6. Statistical Analysis

Calculations were performed using the GraphPad Prism version 9 software (GraphPad Co., San Diego, CA, USA). The normality of distribution was assessed by means of the Shapiro–Wilk test. As all values were normally distributed, the one-way ANOVA test was further applied. The results of statistical analyses were considered significant if they produced *p*-values < 0.05.

## 4. Conclusions

Using in silico studies and in vitro laboratory experiments, we proved that dioscin has a number of favorable therapeutic parameters against *H. pylori*, including antimicrobial activity, ability to interfere with biofilm formation under stationary and flow conditions, and additive enhancement of antibacterial capacity of all three commonly used antibiotics (clarithromycin, metronidazole, and levofloxacin). Based on these results, we suggest that dioscin may be an interesting candidate for new therapies targeting *H. pylori* survivability and virulence.

## Figures and Tables

**Table 1:** Virtual screening hits, their binding free energies, and theoretical inhibition constants (at T = 310.15 K).

Compound	Class	ΔG [kcal/mol]	*K*_i_ [μM]
Dioscin	Saponine glycoside	−9.8	0.122
Zeaxanthin	Carotenoid	−8.9	0.527
Glycyrrhizic acid	Saponine glycoside	−8.8	0.620
Ginsenoside Rg3	Saponine glycoside	−8.6	0.858
Robinin	Flavone glycoside	−8.6	0.858
Amentoflavone	Biflavonoid	−8.5	1.009
Hypericin	Naphthodianthrone	−8.5	1.009
Procyanidin A2	Proanthocyanidin	−8.5	1.009
Procyanidin C1	Proanthocyanidin	−8.2	1.642
Aescin	Saponine glycoside	−8.2	1.642

The inhibition constants were obtained with the Autodock Vina software as described in the Section 3.1.3.

**Table 2:** Minimal inhibitory concentration (MIC) values of 10 selected compounds of natural origin against *H. pylori* J99.

Tested Compounds	MIC [µg/mL]
Dioscin	64
Hypericin	128
Amentoflavone	128
Aescin	256
Glycyrrhetinic acid	>256
Ginsenoside Rg3	>256
Procyanidin A2	>256
Procyanidin C1	>256
Robinin	>256
Zeaxanthin	>256

**Table 3:** Numerical values showing interactions between dioscin and the three classically used antibiotics (clarithromycin [CLR], metronidazole [MTZ], and levofloxacin [LEV]) in antibacterial activity against *H. pylori* J99. Interactions were determined using the checkerboard assay.

Tested Combination	MIC [µg/mL]						FICI (Outcome)
	Dioscin			Antibiotic			
	Alone	Combination	Fold Change	Alone	Combination	Fold Change	
Dioscin + CLR	64	32	2	0.025	0.006	4	0.75 (additive)
Dioscin + MTZ	64	16	4	1	0.5	2	0.75 (additive)
Dioscin + LEV	64	32	2	1	0.5	2	1.0 (additive)

The fractional inhibitory concentration index (FICI) values of ≤ 0.5, > 0.5 to ≤ 1, and > 1 to ≤ 4 were expressed as synergistic, additive, and neutral, respectively.

## Supplementary Materials

Table S1: List of phytochemicals tested by virtual screening with their binding free energies required to attach to the active site of the SpoT protein of H. pylori; Table S2. Results of literature search showing a low or acceptable toxicity profile of phytochemicals selected for testing in the current study [97,98,99,100,101,102,103,104,105,106,107,108]; Figure S1: Representative photos presenting bacterial morphology observed after a 72-h exposure of *H. pylori* J99 to the tested compounds; Figure S2: Representative photos obtained during time-lapse microscopy recordings from experiments determining anti-biofilm activity of dioscin under flow conditions against *H. pylori* J99.

## Sample Availability

Samples of the compounds dioscin, aescin, glycyrrhetinic acid, ginsenoside Rg3, procyanidin A2 and C1, hypericin, robinin, amentoflavone, and zeaxanthin are available from Zbigniew Sroka (Department of Pharmacognosy and Herbal Medicines, Wroclaw Medical University. Poland).
