# Supplementary material for: In Silico Screening and In Vitro Assessment of Natural Products with Anti-Virulence Activity against Helicobacter pylori
Source: Molecules. 2021 Dec 21;27(1):20. doi: 10.3390/molecules27010020 (PMC8746548; doi:10.3390/molecules27010020)
Supplement: Supplementary file 1 [file molecules-27-00020-s001.zip › molecules-1490148-supplementary.pdf]

molecules

MDPI

Supplementary Materials

In silico screening and in vitro assessment of natural products with anti–virulence activity against *Helicobacter pylori*

Maciej Spiegel [1,†,*], Paweł Krzyżek [2,†,*], Ewa Dworniczek [2], Ryszard Adamski [3] and Zbigniew Sroka [1]

1 Department of Pharmacognosy and Herbal Medicines, Wroclaw Medical University, Borowska 211A, 50-556 Wroclaw, Poland
2 Department of Microbiology, Faculty of Medicine, Wroclaw Medical University, Chalubinskiego 4, 50-368 Wroclaw, Poland
3 Laboratory of Microscopic Techniques, Institute of Genetics and Microbiology, University of Wroclaw, Przybyszewskiego 63, 50-001 Wroclaw, Poland

* Correspondence: maciej.spiegel@student.umed.wroc.pl (M.S.), krojcerpawel@gmail.com (P.K.)
† Those authors contributed equally to the paper

Table S1. List of phytochemicals tested by virtual screening with their binding free energies (ΔG [kcal/mol]) required to attach to the active site of the SpoT protein of *H. pylori*.

Tested phytochemical	ΔG	Tested phytochemical	ΔG	Tested phytochemical	ΔG
ailanthone	-8,1	amentoflavone	-8,5	madecassoside	10,6
fortunellin	-8,1	hypericin	-8,5	dioscin	-9,8
naringin	-8,1	lanatoside_B	-8,5	alpha-chaconine	-9,6
tiliroside	-8,1	hinokiflavone	-8,4	alpha-solasonine	-9,5
ruscogenin	-8,1	solanidine	-8,3	rebaudioside_A	-9,5
chrysanthellin_B	-8,1	cupressuflavone	-8,3	alpha-solanine	-9,4
digitoxin	-8,1	diosgenin	-8,3	alpha-solamargine	-9,3
friedelin	-8,1	asiaticoside	-8,3	gracillin	-9,1
lanatoside_C	-8,1	gypenoside_XVII	-8,3	zeaxanthin	-8,9
kaempferol-7-O-neohesperidoside	-8,0	K-strophanthoside	-8,3	sennidine_A	-8,9
rutin	-8,0	procyanidin_A1	-8,2	chrysanthellin_A	-8,9
sennoside_A	-8,0	procyanidin_C1	-8,2	hederacoside_C	-8,9
sennoside_B	-8,0	diosmin	-8,2	sennidine_B	-8,8
sarsasapogenin	-8,0	narirutin	-8,2	glycyrrhizic_acid	-8,8
corosolic_acid	-8,0	neohesperidin	-8,2	gamma-chaconine	-8,7
echinocystic_acid-3-O-glucoside	-8,0	rhoifolin	-8,2	protopine	-8,7
euphol	-8,0	sciadopitysin	-8,2	linarin	-8,7
gitoxin	-8,0	18-beta-glycyrrhetinic_acid	-8,2	didymin	-8,6
lupenone	-8,0	alpha-hederin	-8,2	eriocitrin	-8,6
tomatidine	-7,9	digoxin	-8,2	robinin	-8,6
ginkgolide_B	-7,9	escin	-8,2	protohypericin	-8,6
hesperidin	-7,9	ginsenoside_Rg2	-8,2	ginsenoside_Rb3	-8,6
kaempferol-3-O-rutinoside	-7,9	proscillaridin_A	-8,2	ginsenoside_Rg3	-8,6
vitexin-2''-O-rhamnoside	-7,9	pseudoginsenoside_F11	-8,2	tomatine	-8,5
neoruscogenin	-7,9	procyanidin_B2	-8,1	procyanidin_A2	-8,5
caulophyllogenin	-7,9				

calendoflavoside	-7,3	ginsenoside_Rg1	-7,6	delphinidin-3-O-sambubioside	-7,8
eupatolin	-7,3	sanguinarine	-7,5	pelargonidin-3-O-rutinoside	-7,8
gossypetin-8-O-glucuronide	-7,3	solasodine	-7,5	7,8-benzoflavone	-7,8
luteolin-7-O-glucoside	-7,3	tetrahydroalstonine	-7,5	isorhoifolin	-7,8
neoeriocitrin	-7,3	procyanidin_B1	-7,5	liquiritin	-7,8
ononin	-7,3	baicalin	-7,5	luteolin-4'-O-glucoside	-7,8
peltatoside	-7,3	icariin	-7,5	quercetin-3-O-glucuronide	-7,8
bilobalide	-7,3	isovitexin	-7,5	stigmasterol	-7,8
nootkatone	-7,3	quercetin-7-O-glucoside	-7,5	trillin	-7,8
3-O-acetyl-beta-boswellic_acid	-7,3	schaftoside	-7,5	beta-amyrin	-7,8
digitoxigenin	-7,3	spiraeoside	-7,5	ginsenoside_Rb2	-7,8
notoginsenoside_R1	-7,3	trans-epsilon-viniferin	-7,5	lupeol	-7,8
quillaic_acid	-7,3	tanshinone_IIA	-7,5	maslinic_acid	-7,8
ursolic_acid_methylester	-7,3	beta-boswellic_acid	-7,5	uvaol	-7,8
ellipticine	-7,2	cucurbitacin_I	-7,5	stevioside	-7,7
sempervirine	-7,2	echinocystic_acid	-7,5	luteolin-3',7-di-O-glucoside	-7,7
beta-cryptoxanthin	-7,2	ginsenoside_CK	-7,5	orientin	-7,7
andrographolide	-7,2	ginsenoside_Rd	-7,5	poncirin	-7,7
ginkgolide_J	-7,2	lupeol_acetate	-7,5	salvianolic_acid_B	-7,7
avicularin	-7,2	madecassic_acid	-7,5	frangulin_A	-7,7
genistein-7-O-glucuronide	-7,2	protopanaxadiol	-7,5	11beta,13-dihydrolactucopicrin	-7,7
kaempferol-3-O-rhamnoside	-7,2	ursolic_acid	-7,5	11-keto-beta-boswellic_acid	-7,7
neodiosmin	-7,2	senkirkine	-7,4	18-alpha-glycyrrhetinic_acid	-7,7
alpha-tocotrienol	-7,2	flavanomarein	-7,4	beta-amyrin_acetate	-7,7
amarogentin	-7,2	glabridin	-7,4	betulinic_acid	-7,7
stachyose	-7,2	guaijaverin	-7,4	cucurbitacin_D	-7,7
amygdalin	-7,2	hesperetin-7-O-glucoside	-7,4	cucurbitacin_E	-7,7
hamamelitannin	-7,2	isoschaftoside	-7,4	erythrodiol	-7,7
3,5-di-O-caffeoyl_quinic_acid	-7,2	quercetin-3,4'-di-O-glucoside	-7,4	ginsenoside_Rf	-7,7
maritimein	-7,2	vitexin	-7,4	oleanolic_acid	-7,7
bulnesol	-7,2	nuzhenide	-7,4	GTP_B	-7,6
guaiazulene	-7,2	trifolirhizin	-7,4	conessine	-7,6
guaiol	-7,2	3-O-acetyl-11-keto-beta-boswellic_acid	-7,4	keracyanin	-7,6
cucurbitacin_B	-7,2	alpha-boswellic_acid	-7,4	gallocatechin_gallate	-7,6
ginsenoside_Rh2	-7,2	medicagenic_acid	-7,4	procyanidin_B3	-7,6
GTP_C	-7,1	oleanolic_acid_methylester	-7,4	datiscin	-7,6
chelidonine	-7,1	syrosingopine	-7,3	eriodictyol-7-O-glucoside	-7,6
yohimbic_acid	-7,1	cyanidin-3-O-sambubioside-5-O-glucoside	-7,3	isorhamnetin-3-O-rutinoside	-7,6
yohimbine	-7,1	cyanidin-3-O-sophoroside	-7,3	kaempferol-7-O-glucoside	-7,6
callistephin	-7,1	delphin	-7,3	puerarin	-7,6
bavachin	-7,1	ideain	-7,3	harpagoside	-7,6
daidzin	-7,1	myrtillin	-7,3	alpha-amyrin	-7,6
diosmetin-7-O-glucoside	-7,1	epicatechin_gallate	-7,3	ginsenoside_Rc	-7,6

beta-tocotrienol	-6,8	ajmalicine	-6,9	glycitin	-7,1
agnuside	-6,8	gelsemine	-6,9	gossypin	-7,1
trilobatin	-6,8	cyanin	-6,9	homoorientin	-7,1
beta-sitosterol	-6,8	malvin	-6,9	silybin	-7,1
aloin_B	-6,8	pelargonin	-6,9	garcinoic_acid	-7,1
11beta,13-dihydrolactucin	-6,8	ginkgolide_C	-6,9	cynarin	-7,1
mangiferin	-6,8	2'-methoxyflavone	-6,9	isoliquiritin	-7,1
seneciphylline	-6,7	genistin	-6,9	leontopodic_acid	-7,1
capsanthin	-6,7	kaempferol-3-O-glucoside	-6,9	polydatin	-7,1
bergamottin	-6,7	karanjin	-6,9	sesamin	-7,1
wedelolactone	-6,7	luteolin	-6,9	frangulin_B	-7,1
3-hydroxyflavone	-6,7	myricetin-3-O-glucoside	-6,9	alantolactone	-7,1
7-hydroxy-5-methylflavone	-6,7	quercitrin	-6,9	3-O-acetyl-alpha-boswellic_acid	-7,1
gossypetin	-6,7	gluconasturtiin	-6,9	betulin	-7,1
myricetin-3-O-galactoside	-6,7	delta-tocotrienol	-6,9	betulin_diacetate	-7,1
syringetin	-6,7	marein	-6,9	cymarin	-7,1
glucoerucin	-6,7	rhapontin	-6,9	elemolic_acid	-7,1
glucoraphanin	-6,7	cholesterol	-6,9	ginsenoside_Rb1	-7,1
catalpol	-6,7	fucosterol	-6,9	hederagenin	-7,1
gentianose	-6,7	aloin_A	-6,9	chelerythrine	-7,0
phloretic_acid	-6,7	dihydroartemisinin	-6,9	corynanthine	-7,0
bisdemethoxycurcumin	-6,7	trans-caryophyllene	-6,9	emetine	-7,0
chicoric_acid	-6,7	cycloartenol_acetate	-6,9	rauwolscine	-7,0
ellagic_acid	-6,7	cyanidin	-6,8	lutein	-7,0
aromadendrene	-6,7	apocarotenal	-6,8	catechin_gallate	-7,0
picrotin	-6,7	beta-carotene	-6,8	ginkgolide_A	-7,0
berberine	-6,6	marrubiin	-6,8	daidzein-7-O-glucuronide	-7,0
cinchonidine	-6,6	5-methoxyflavone	-6,8	eriodictyol	-7,0
harman	-6,6	7-hydroxyflavone	-6,8	flavone	-7,0
senecionine	-6,6	7-hydroxyflavonol	-6,8	quercetagetin-7-O-glucoside	-7,0
cyanidin-3-O-arabinoside	-6,6	apigenin-7-O-glucoside	-6,8	quercetin-3-O-glucopyranoside	-7,0
delphinidin-3-O-galactoside	-6,6	bavachinin	-6,8	sophoricoside	-7,0
kuromanin	-6,6	hyperoside	-6,8	gamma-tocotrienol	-7,0
lycopene	-6,6	isosakuranetin	-6,8	echinacoside	-7,0
auraptene	-6,6	kaempferol-3-O-glucuronide	-6,8	sesamolin	-7,0
3',4'-dihydroxyflavone	-6,6	myricitrin	-6,8	verbascoside	-7,0
4',7-dihydroxyflavone	-6,6	naringenin-7-O-glucoside	-6,8	carminic_acid	-7,0
4'-hydroxyflavanone	-6,6	pinobanksin	-6,8	alpha-cedrene	-7,0
galangin	-6,6	pinocembrin	-6,8	chamazulen	-7,0
pratol	-6,6	saponarin	-6,8	zerumbone	-7,0
tectochrysin	-6,6	sissotrin	-6,8	poststerone	-7,0
glucobrassicin	-6,6	4-methoxyglucobrassicin	-6,8	asiatic_acid	-7,0
glucomoringin	-6,6	neoglucobrassicin	-6,8	betulinic_acid_methyl_ester	-7,0

demethoxycurcumin	-6,4
isoliquiritigenin	-6,4
sieboldin	-6,4
emodin	-6,4
alpha-santonin	-6,4
beta-curcumene	-6,4
protopanaxatriol	-6,4
ATP_C	-6,3
ATP_B	-6,3
monocrotalin	-6,3
delphinidin	-6,3
malvidin	-6,3
robinetinidin	-6,3
epigallocatechin	-6,3
4-methylumbelliferone	-6,3
7-methylcoumarin	-6,3
coumestrol_dimethyl_ether	-6,3
dalbergin	-6,3
esculin	-6,3
imperatorin	-6,3
3',4',7,8-tetramethoxyflavone	-6,3
3',4'-dimethoxyflavone	-6,3
3,6-dihydroxyflavone	-6,3
3,7-dihdyroxy-3',4',5'-trimethoxyflavone	-6,3
4'-methoxyflavanone	-6,3
6-hydroxyflavanone	-6,3
7-methoxyflavonol	-6,3
acacetin	-6,3
diosmetin	-6,3
hesperetin	-6,3
homoeriodictyol	-6,3
isorhamnetin-3-O-glucoside	-6,3
isorhamnetin	-6,3
quercetagetin	-6,3
sinensetin	-6,3
syringetin-3-O-galactoside	-6,3
tangeretin	-6,3
tricetin_pentamethyl_ether	-6,3
glucolimnanthin	-6,3
gluconapin	-6,3
azulene	-6,3
gamma-terpinene	-6,3
limonene	-6,3
luteolinidin	-6,4
oenin	-6,4
catechin	-6,4
gallocatechin	-6,4
coumestrol	-6,4
esculetin	-6,4
3',4',7,8-tetrahydroxyflavone	-6,4
3',4',7-trihydroxyflavone	-6,4
5,7-dihydroxy-3',4',5'-trimethoxyflavone	-6,4
5,7-dimethoxyflavanone	-6,4
5-methyl-7-methoxyisoflavone	-6,4
6-hydroxyflavone	-6,4
6-methoxyluteolin	-6,4
7,8-dihydroxyflavone	-6,4
7-hydroxyflavanone	-6,4
7-methoxyflavone	-6,4
baicalein	-6,4
datiscetin	-6,4
flavanone	-6,4
geraldol	-6,4
herbacetin	-6,4
kaempferide	-6,4
kaempferol	-6,4
naringenin	-6,4
pinocembrin-7-methylether	-6,4
quercetin	-6,4
syringetin-3-O-glucoside	-6,4
tamarixetin	-6,4
taxifolin	-6,4
tricetin	-6,4
glucoraphenin	-6,4
alpha-tocopherol	-6,4
hastatoside	-6,4
menthofuran	-6,4
shanzhiside_methyl_ester	-6,4
verbenalin	-6,4
vulpic_acid	-6,4
4'-hydroxychalcone	-6,4
4'-methoxychalcone	-6,4
4,4'-dimethoxychalcone	-6,4
4-hydroxychalcone	-6,4
4-methoxychalcone	-6,4
chlorogenic_acid	-6,4
glucotropaeolin	-6,6
sinalbin	-6,6
delta-amplexichromanol	-6,6
2-hydroxychalcone	-6,6
deoxyrhapontin	-6,6
phloridizin	-6,6
podophyllotoxin	-6,6
usnic_acid	-6,6
anthraquinone	-6,6
cedrol	-6,6
parthenolide	-6,6
ginsenoside_Rh1	-6,6
atropine	-6,5
hyoscyamine	-6,5
piperine	-6,5
fraxin	-6,5
carnosol	-6,5
3-methoxyflavone	-6,5
5-hydroxyflavone	-6,5
5-methoxyflavanone	-6,5
6-methoxyflavanone	-6,5
6-methoxyflavone	-6,5
apigenin	-6,5
baicalein-7-methylether	-6,5
chrysin	-6,5
chrysin_dimethyl_ether	-6,5
chrysoeriol	-6,5
liquiritigenin	-6,5
myricetin	-6,5
scutellarein	-6,5
wogonin	-6,5
13'-carboxy-alpha-tocopherol	-6,5
4-cymene	-6,5
carvacrol	-6,5
harpagide	-6,5
loganic_acid	-6,5
evernic_acid	-6,5
chalcone	-6,5
neohesperidin_dihydrochalcone	-6,5
sulfuretin	-6,5
ATP_A	-6,4
ajmaline	-6,4
laudanosoline	-6,4

2'-hydroxyflavanone	-6,0	magnolol	-6,2	sabinene	-6,3
3',4',7-trihydroxyisoflavone	-6,0	aloe-emodin	-6,2	terpinolene	-6,3
5,7-dihydroxy-3',4',5'-trimethoxyflavanone	-6,0	rhein	-6,2	trehalose	-6,3
eupatorin-5-methylether	-6,0	alpha-curcumene	-6,2	picein	-6,3
fustin	-6,0	longifolene	-6,2	2',6'-dihydroxy-4,4'-dimethoxychalcone	-6,3
genistein-4',7-dimethylether	-6,0	palmatine	-6,1	neochlorogenic_acid	-6,3
glycitein	-6,0	apigeninidin	-6,1	rosmarinic_acid	-6,3
irisflorentin	-6,0	psoralen	-6,1	salidroside	-6,3
kaempferol_3,7,4'-trimethyl_ether	-6,0	kirenol	-6,1	alizarin	-6,3
nobiletin	-6,0	7,8-dimethoxyflavone	-6,1	chrysophanol	-6,3
prunetin	-6,0	baicalein-5,6,7-trimethylether	-6,1	lapachol	-6,3
quercetin-3,7,3',4'-tetramethyl_ether	-6,0	biochanin_A	-6,1	alpha-bisabolol	-6,3
quercetin-3-methylether	-6,0	genistein	-6,1	cynaropicrin	-6,3
resokaempferol	-6,0	genkwanin	-6,1	quinidine	-6,2
glucoiberin	-6,0	quercetin_3,4'-dimethyl_ether	-6,1	vincamine	-6,2
3-carene	-6,0	rhamnazin	-6,1	epicatechin	-6,2
isomenthol	-6,0	rhamnetin	-6,1	carnosic_acid	-6,2
butein	-6,0	sakuranetin	-6,1	forskolin	-6,2
flavokawain_A	-6,0	glucoberteroin	-6,1	apigenin-4',5,7-trimethylether	-6,2
hesperetin_dihydrochalcone-4'-O-glucoside	-6,0	delta-tocopherol	-6,1	dihydromyricetin	-6,2
syringin	-6,0	2-carene	-6,1	dihydrorobinetin	-6,2
flavokawain_B	-6,0	alpha-terpinene	-6,1	equol	-6,2
myricanol	-6,0	aucubin	-6,1	eupatorin	-6,2
1,8-dihydroxyanthraquinone	-6,0	cuminaldehyde	-6,1	fisetin	-6,2
plumbagin	-6,0	oleuropein	-6,1	ipriflavone	-6,2
acetoxyvalerenic_acid	-6,0	R-pulegone	-6,1	isoxanthohumol	-6,2
fisetinidin	-5,9	alpha-sophorose	-6,1	morin	-6,2
pelargonidin	-5,9	3-hydroxybenzoic_acid	-6,1	robinetin	-6,2
bergaptol	-5,9	aberchrome_540	-6,1	glucocheirolin	-6,2
coumarin	-5,9	3-coumaric_acid	-6,1	beta-tocopherol	-6,2
3',4',5,5',6,7-hexamethoxyflavone	-5,9	chlorogenic_acid_methylester	-6,1	gentiopicroside	-6,2
6,7,4'-trihydroxyisoflavone	-5,9	curcumin	-6,1	loganin	-6,2
quercetin-3,5,7,3',4'-pentamethylether	-5,9	flavokawain_C	-6,1	piperitone	-6,2
scutellarein_tetramethyl_ether	-5,9	homobutein	-6,1	sweroside	-6,2
glucoarabin	-5,9	hypophyllanthin	-6,1	beta-gentiobiose	-6,2
glucolepidiin	-5,9	yangonin	-6,1	DL-kawain	-6,2
gamma-tocopherol	-5,9	physcion	-6,1	ligustilide	-6,2
ajugol	-5,9	(3aR)-sclareolide	-6,1	2'-hydroxychalcone	-6,2
alpha-thujone	-5,9	GTP_A	-6,0	2,3-dimethoxy-2'-hydroxychalcone	-6,2
carvacryl_acetate	-5,9	boldine	-6,0	3,4-dimethoxychalcone	-6,2
geniposide	-5,9	quinine	-6,0	resveratrol	-6,2
isopulegol	-5,9	guibourtinidin	-6,0	xanthohumol	-6,2
		angelicin	-6,0	honokiol	-6,2

gallic_acid	-5,3	nordihydroguaiaretic_acid	-5,7	menthyl_acetate	-5,9
methylgallate	-5,3	juglone	-5,7	R-alpha-phellandrene	-5,9
phloroglucinol	-5,3	4-methoxycoumarin	-5,6	valtrate	-5,9
4-coumaric_acid	-5,3	6,7-dihydroxy-4-methylcoumarin	-5,6	3,4-dihydroxyacetophenone	-5,9
cinnamic_acid_ethyl_ester	-5,3	6-hydroxycoumarin	-5,6	4-(4-hydroxyphenyl)-2-butanone	-5,9
cinnamic_acid_methyl_ester	-5,3	7-ethoxycoumarin	-5,6	4-hydroxybenzoic_acid	-5,9
trans-cinnamic_acid	-5,3	citropten	-5,6	ethyl-4-hydroxybenzoate	-5,9
khellin	-5,3	herniarin	-5,6	hydroxytyrosol	-5,9
convolvamine	-5,2	isopimpinellin	-5,6	eriodictyol_chalcone	-5,9
daphnetin-7-methylether	-5,2	isoscopoletin	-5,6	hesperetin_dihydrochalcone	-5,9
daphnetin	-5,2	luteolin_tetramethylether	-5,6	piceatannol	-5,9
chrysanthemyl_alcohol	-5,2	1,4-cineole	-5,6	lawsone	-5,9
4-O-methylgallic_acid	-5,2	alpha-terpineol	-5,6	3,4-dihydrocoumarin	-5,8
catechol	-5,2	beta-pinene	-5,6	4-methyl-7-ethoxycoumarin	-5,8
methyl-4-hydroxybenzoate	-5,2	chrysanthemolactone	-5,6	bergapten	-5,8
fraxetin	-5,1	terpinen-4-ol	-5,6	isobergapten	-5,8
fraxidin	-5,1	D-saccharose	-5,6	umbelliferone	-5,8
estragole	-5,1	alpha-ionone	-5,6	xanthotoxol	-5,8
lavandulyl_acetate	-5,1	D-salicin	-5,6	4',6,7-trimethoxyisoflavone	-5,8
linalool	-5,1	protocatechualdehyde	-5,6	casticin	-5,8
myrcene	-5,1	cinnamyl_acetate	-5,6	daidzein	-5,8
acetoisovanillone	-5,1	tetrahydro-curcumin	-5,6	gardenin_A	-5,8
acetovanillon	-5,1	arbutin	-5,6	glucobrassicanapin	-5,8
homogentisic_acid	-5,1	carophyllene_oxide	-5,6	carvone	-5,8
myristicin	-5,1	farnesol	-5,6	phloretin	-5,8
2-coumaric_acid	-5,1	dihydrocapsaicin	-5,5	valerenic_acid	-5,8
3,4,5-trihydroxycinnamic_acid	-5,1	sparteine	-5,5	cytisine	-5,7
ferulic_acid	-5,1	5,7-dihydroxy-4-methylcoumarin	-5,5	piperlongumine	-5,7
ferulic_acid_methyl_ester	-5,1	scopoletin	-5,5	3-hydroxycoumarin	-5,7
phyllanthin	-5,1	sabinyl_acetate	-5,5	4-hydroxycoumarin	-5,7
sinapic_acid	-5,1	thymol	-5,5	5-geranyloxy-7-methoxycoumarin	-5,7
trans-nerolidol	-5,1	5-hydroxy-L-tryptophan	-5,4	osthol	-5,7
procaine	-5,0	6,7-dimethylesculetin	-5,4	xanthotoxin	-5,7
tyramine	-5,0	alpha-pinene	-5,4	4',7-dimethoxyisoflavone	-5,7
camphene	-5,0	isomenthone	-5,4	formononetin	-5,7
isobornyl_acetate	-5,0	verbenone	-5,4	epiprogoitrin	-5,7
myrtenol	-5,0	ethylgallate	-5,4	progoitrin	-5,7
trans-myrtanol	-5,0	visnagin	-5,4	13'-hydroxy-alpha-tocopherol	-5,7
D-glucose	-5,0	nonivamide	-5,3	menthol	-5,7
D-mannose	-5,0	physalien	-5,3	beta-damascone	-5,7
4-hydroxybenzoic_acid_propyl_ester	-5,0	fenchone	-5,3	dhurrin	-5,7
6-shogaol	-5,0	genipin	-5,3	helicin	-5,7
8-gingerol	-5,0	3-O-methylgallic_acid	-5,3	trans-cinnamaldehyde	-5,7

linoleic_acid	-4,6	dillapiole	-4,9	acetyleugenol	-5,0
oleic_acid	-4,6	caffeine	-4,8	alpha-asarone	-5,0
borneol	-4,6	synephrine	-4,8	eugenol	-5,0
isoborneol	-4,6	camphor	-4,8	eugenol_methylether	-5,0
linalyl_acetate	-4,6	citral	-4,8	homovanillic_acid	-5,0
D-fructose	-4,6	citronellol	-4,8	quinic_acid	-5,0
D-xylose	-4,6	eucalyptol	-4,8	tyrosol	-5,0
L-arabinose	-4,6	lavandulol	-4,8	caffeic_acid	-5,0
vanillin	-4,6	nerol	-4,8	caffeic_acid_ethyl_ester	-5,0
embelin	-4,6	acetosyringon	-4,8	coniferyl_alcohol	-5,0
geraniol	-4,5	gentisic_acid	-4,8	theophylline	-4,9
beta-asarone	-4,5	salicylic_acid_methylester	-4,8	alpha-tocopherol_acetate	-4,9
pseudopelletierine	-4,4	syringic_acid	-4,8	bornyl_acetate	-4,9
linolenic_acid	-4,4	dihydrocaffeic_acid	-4,8	citronellal	-4,9
gamma-nonalactone	-4,4	dihydroconiferyl_alcohol	-4,8	citronellyl_acetate	-4,9
L-arginine	-4,3	isoferulic_acid	-4,8	geranyl_acetate	-4,9
gamma-undecalactone	-4,3	theobromine	-4,7	D-galactose	-4,9
L-theanine	-4,2	petroselinic_acid	-4,7	L-rhamnose	-4,9
palmitic_acid	-4,2	10-gingerol	-4,7	2,3-dihydroxybenzoic_acid	-4,9
Betonicine	-4,1	alpha-resorcylic_acid	-4,7	2-phenylethanol	-4,9
L-alliin	-4,1	benzyl_alcohol	-4,7	3,4-dihydroxyphenylacetic_acid	-4,9
stachydrine	-4,1	isovanillin	-4,7	4-hydroxybenzaldehyde	-4,9
myristic_acid	-4,1	dihydrosinapyl_alcohol	-4,7	4-hydroxyphenylacetic_acid	-4,9
3-octanone	-4,0	apiole	-4,7	6-gingerol	-4,9
S-allyl-L-cysteine	-3,9	lupinine	-4,6	protocatechuic_acid	-4,9
		trigonelline	-4,6	vanillic_acid	-4,9

Table S2. Results of literature search showing a low or acceptable toxicity profile of phytochemicals selected for testing in the current study.

Selected compound	Reference(s) indicating acceptable toxicity profile
Dioscin	[97,98]
Hypericin	[99,100]
Amentoflavone	[101]
Aescin	[102]
Glycyrrhetinic acid	[103]
Ginsenoside Rg3	[104]
Procyanidin A2	[105]
Procyanidin C1	[105]
Robinin	[106]
Zeaxanthin	[107,108]

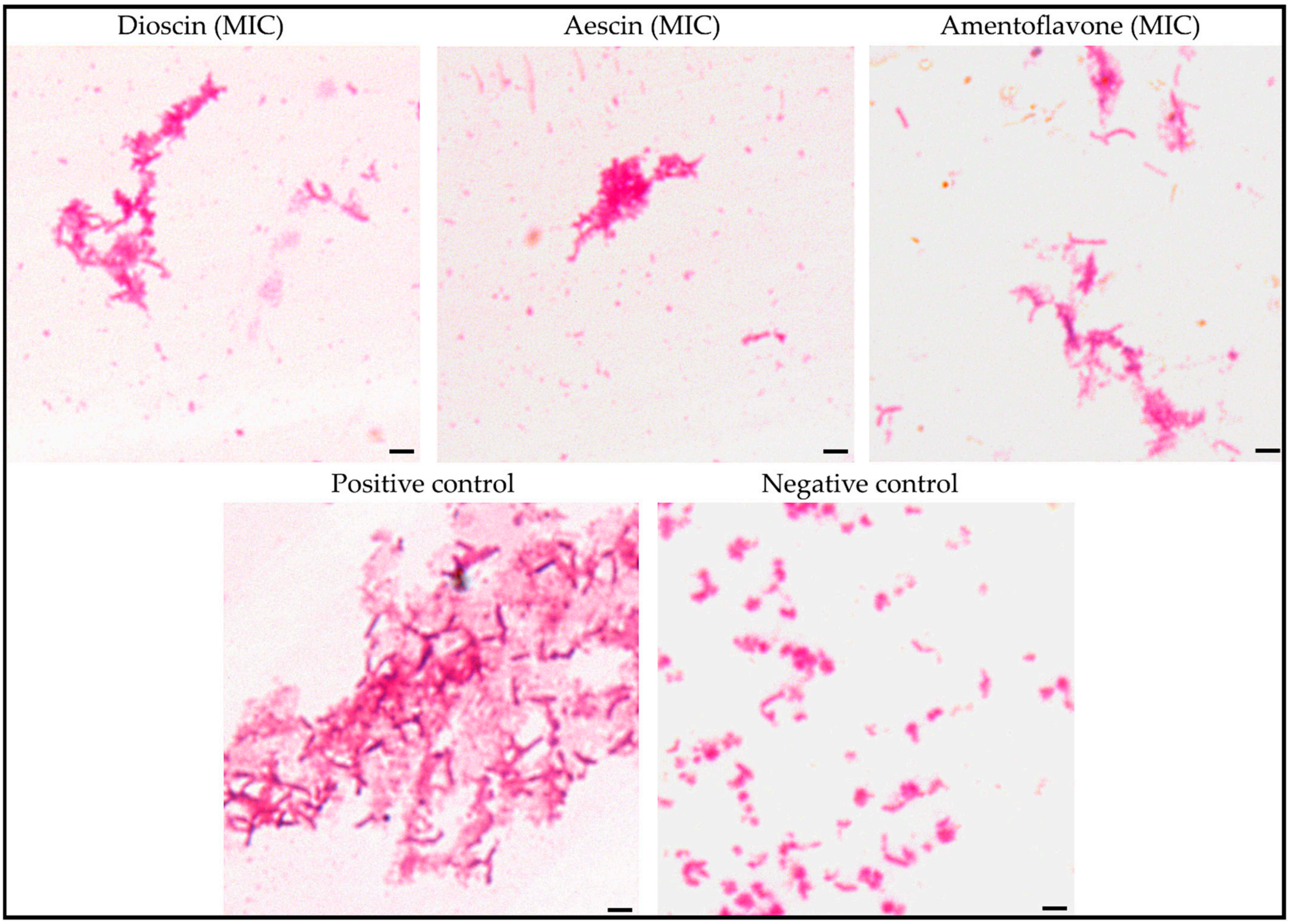

Figure S1. Representative photos presenting bacterial morphology observed after a 72-h exposure of *H. pylori* J99 to the tested compounds (dioscin (64 μg/mL), aescin (256 μg/mL), or amentoflavone (128 μg/mL)). Positive and negative control samples were bacteria not exposed to any antimicrobial and treated with minimal inhibitory concentration of amoxicillin (0.06 μg/mL), respectively. The elongated cylindrical forms are bacteria of the rod/spiral shape, while tiny spherical cells present coccoid forms. Scale bars show 2 μm.

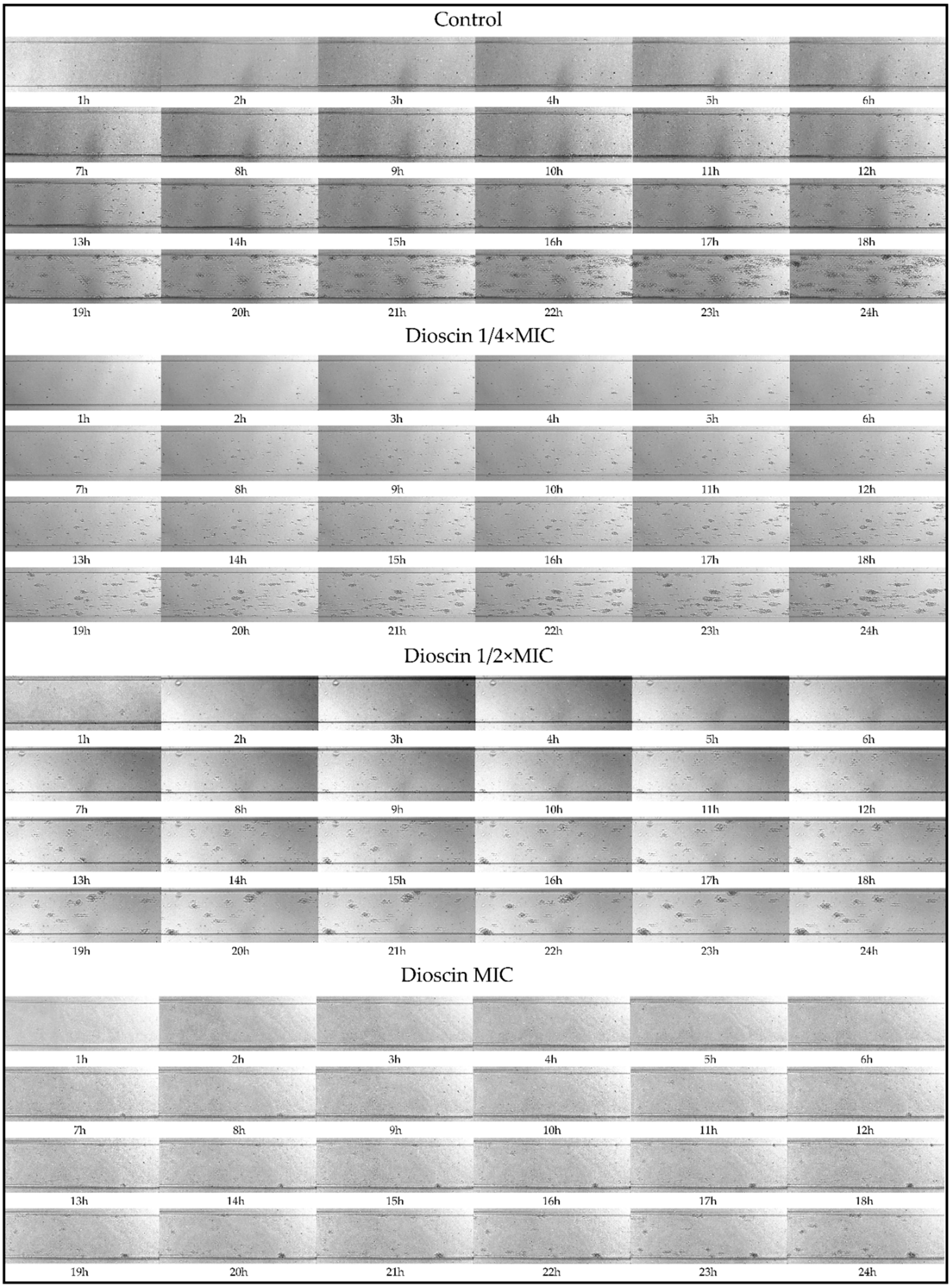

Figure S2. Representative photos obtained during time-lapse microscopy recordings from experiments determining anti-biofilm activity of dioscin under flow conditions against *H. pylori* J99. The flow conditions were generated using the Bioflux 1000 system. The MIC, ½×MIC and ¼×MIC had values of 64 μg/mL, 32 μg/mL, and 16 μg/mL, respectively. Control samples were bacteria not exposed to any concentration of dioscin.
